# Short-Term Effects and Early Complications of Balloon-Occluded Retrograde Transvenous Obliteration for Gastric Varices

**DOI:** 10.5402/2012/919371

**Published:** 2012-12-05

**Authors:** Manabu Watanabe, Kazue Shiozawa, Takashi Ikehara, Shigeru Nakano, Michio Kougame, Takafumi Otsuka, Yoshinori Kikuchi, Koji Ishii, Yoshinori Igarashi, Yasukiyo Sumino

**Affiliations:** Division of Gastroenterology and Hepatology, Department of Internal Medicine, Toho University Medical Center, Omori Hospital, 6-11-1 Omorinishi, Ota-ku, Tokyo 143-8541, Japan

## Abstract

The short-term effects of balloon-occluded retrograde transvenous obliteration (BRTO) to treat gastric varices were evaluated by using computed tomography (CT) and gastroscopy (GF). The subjects were 77 patients who underwent BRTO to treat gastric varices. The short-term effects of BRTO were investigated with regard to ascites, pleural effusion, venous thrombus, and esophageal varices by comparing the findings of CT and GF performed within one month before and after BRTO. The mean duration of followup was 960.1 days. Ascites and pleural effusion were exacerbated after BRTO in 26 (33.8%) and 31 (40.3%), respectively. A significant difference in ascites exacerbation was noted in patients with hypoalbuminemia and a high Child-Pugh score, and a significant difference in exacerbation of pleural effusion was noted in patients with hypoalbuminemia. Venous thrombus was noted in 7 patients (9.1%). Esophageal varices were exacerbated in 14 (21.2%) of the 66 patients. The 2-year survival rate was 720 days, and significant differences were noted in the Child-Pugh classification and the concomitance of hepatocellular carcinoma (HCC) on multivariate analysis of prognosis-related factors. *Conclusion*. The frequencies of exacerbation of ascites, pleural effusion, and esophageal varices after BRTO were high but these may not be related to survival.

## 1. Introduction

Balloon occluded retrograde transvenous obliteration (BRTO) is widely accepted as an effective treatment for gastric varices [1, 2]. Sclerosing agents, that is, ethanolamine oleate (EO) and 50% glucose, are injected directly into varices via the drainage veins, and at the same time, blood flow in the varices is stopped by retrograde balloon occlusion. This method can achieve a high-BRTO success rate, >90%, and a low-recurrence rate, <10% [1–4]. However, BRTO also markedly influences portal hemodynamics because it occludes major portosystemic shunts, it increases the portal hepatic blood flow and portal blood pressure. In many reports, the liver function has been shown to be improved by an increase in portal hepatic blood flow [5, 6], but BRTO may also exacerbate symptoms of portal hypertensive changes, including ascites, splenomegaly, and the development of portosystemic collaterals, and the influence on respiratory function and complication of venous thrombus are of concern [7–12]. While aggravation of esophageal varices in the late period or long term by BRTO has been investigated [13], the short-term effects and complications after BRTO in patients with portal hypertension have not been investigated sufficiently, to date. In this study, we retrospectively evaluated the short-term effects and early complications of BRTO by performing computed tomography (CT) and gastroscopy (GF) before and after BRTO and extracted the predictors of the cumulative survival rate by multivariate analysis.

## 2. Methods

### 2.1. Patients

Among the hepatic cirrhosis and portal hypertensive patients with gastrorenal shunt, 79 underwent BRTO to treat gastric varices between October 2006 and July 2011, and 77 of them were involved in this study, after excluding 2 patients who could not be followed after BRTO (Table 1). This study was approved by the IRB of our hospital. There were 51 male and 26 female patients (age: 14–90 years, mean age: 61.6 ± 12.2 years). Emergency BRTO was performed within 48 hours after temporary hemostasis of gastric variceal hemorrhage with GF in 15 patients, prophylactic BRTO for hemorrhage was performed in 62 patients with gastric varices. The background liver disease was viral hepatic cirrhosis in 39 patients, alcoholic hepatic cirrhosis in 26, nonalcoholic steatohepatitis (NASH) in 4, primary biliary cirrhosis (PBC) in 3, autoimmune hepatitis (AIH) in 2, and idiopathic portal hypertension (IPH) in 3. The mean serum albumin (Alb) and total bilirubin (T-bil) levels, prothrombin time (PT), and platelet count immediately before BRTO were 3.2 ± −0.6 (g/dL), 1.3 ± −0.7 (mg/dL), 72.7 ± −13.2 (%), and 9.2 ± −3.7 (×10^4^/*μ*L), respectively. The Child-Pugh score was 7.2 ± −1.7, and the Child-Pugh class was A in 32 patients, B in 37, and C in 8. Thirty patients had hepatocellular carcinoma (HCC). Regarding esophageal varices, GF was not performed within one week before BRTO in 9 patients and within one month after BRTO in 2 patients. Excluding these patients, 66 patients were included in the evaluation.

### 2.2. BRTO Procedure

Written informed consent for BRTO was obtained from all patients or responsible family members. BRTO was performed as reported previously [14–17]. Employing the Seldinger method, a sheath was inserted via the right femoral or right internal jugular vein, and a 5-6 Fr catheter with a 10–30 mm occlusion balloon was inserted into the draining vessel branching from the left renal vein using a guide wire. After advancing the balloon into the draining vessel, pressure was applied to the balloon, and retrograde venography was performed under balloon occlusion. When thin collateral circulation, that is, via the inferior phrenic and pericardiac veins, was imaged, these underwent embolization with a metal coil using a microcatheter. Under balloon occlusion, 5% EO (1 : 1 mixture of 10% ethanolamine oleate and iopamidol contrast medium) was slowly injected into the varix intermittently under fluoroscopy. At the same time, haptoglobin was injected into the median cubital vein. The catheter was left in the vessel with the balloon inflated until the following day and then removed after confirming complete occlusion of the varix under fluoroscopy. When occlusion was incomplete, the 5% EO injection was repeated until complete occlusion was achieved. The mean volume of injected 5% EO was 21.5 ± −8.7 mL. The injection volume of EO was determined based on the volume of contrast medium required to image the varix, and the maximum volume per day was set at 0.4 mL/Kg.

### 2.3. Images and Analysis

CT was performed within 2 weeks after BRTO using one of 2 multirow detector computed tomography (MDCT) scanners (Toshiba Medical Systems and GE healthcare). The images were obtained in the craniocaudal direction from the superior margin of the heart to the lower pole of the right kidney in 5 mm slices with 2.5–5 mm reconstruction. Ninety milliliters of nonionic contrast medium (Ultravist 300, Schering AG, Berlin, Germany) was injected at 3 mL/sec using an automatic injector. Images of the arterial and equilibrium phases were obtained, 35 and 120 seconds after intravenous injection of the contrast medium, respectively. On plain CT and imaging of the equilibrium phase, the region over the pelvis was imaged. Preprocedual CT was performed within one week before BRTO. 

The pre- and postprocedual CT scans were retrospectively compared by two gastroenterologists working in consensus with respect to the short-term effects of BRTO on the following changes; ascites, pleural effusion, and venous thrombus. Regarding the ascites and pleural effusion, new appearance and increases upon comparison of the CT images were judged as exacerbation. As for venous thrombus, those that newly appeared in the portal, splenic, and left renal veins, other than the varices, were regarded as positive. As for esophageal varices, new appearance on GF within one month after BRTO, an increase in form (*F* factor), and appearance of red color sign (RC) were judged as exacerbation. Preoperative GF was performed within one week before BRTO.

Factors influencing ascites, pleural effusion, and venous thrombus after BRTO were investigated. The cumulative survival rate and factors influencing the cumulative survival rate were also investigated. Among the factors influencing the cumulative survival rate, age ≥ 70 years, gender, emergency BRTO, viral hepatic cirrhosis, serum albumin level ≥3 (g/dL), elevated total bilirubin level ≥1.4 (mg/dL), PT of 70% or higher, platelet count of 8.0 (× 10^4^/*μ*L) or higher, Child-Pugh classification, concomitance of HCC, exacerbation of ascites and pleural effusion, and the appearance of venous thrombi were investigated. For blood test findings, those obtained on the day before or the day of BRTO were used. The significance of differences in background factors was analyzed using the paired *t*-, Mann-Whitney, and *χ* ^2^-tests. For analysis of survival, the Kaplan-Meier method and Log rank test were used, and when a significant difference was observed, multivariate analysis using the Cox proportional hazards model was performed, and the hazard ratio was calculated. *P* values of less than 0.05 were considered to indicate statically significance.

## 3. Results

**Figure 1:**
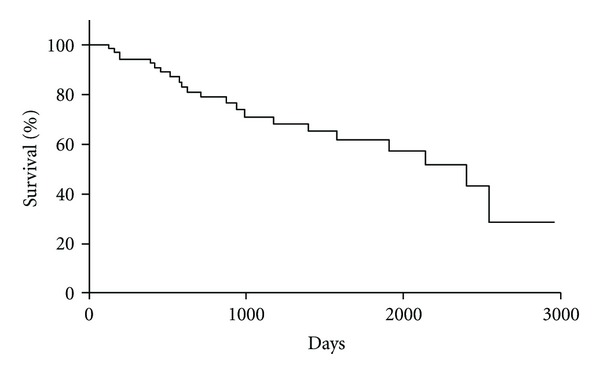
Cumulative survival rate of all patients after BRTO.

Table 2 shows the results of CT and GF after BRTO. Ascites and pleural effusion were exacerbated in 26 (33.8%) and 31 (40.3%) of the 77 patients, respectively. Ascites was present in 11 patients before BRTO, and improved in one. Pleural effusion was exacerbated on the left side in 25 (80.7%) of the 31 patients with exacerbation of pleural effusion. No pleural effusion was present before BRTO in any patient. Venous thrombus was noted in 7 patients (9.1%): portal thrombus in 3, splenic venous thrombus in 2, and left renal venous thrombus in 2. Exacerbation of esophageal varices occurred in 14 (21.2%) of the 66 patients. Among the factors exacerbating ascites, significant differences were noted in patients with hypoalbuminemia (*P* = 0.0067), hyperbilirubinemia (*P* = 0.0342), low PT% (*P* = 0.0076), and high Child-Pugh score (*P* = 0.0023) (Table 3). A significant difference in exacerbation of pleural effusion was noted only in the patients with hypoalbuminemia (*P* = 0.0412) (Table 4). No significant differences were noted due to venous thrombus for any of the factors (Table 5). Simultaneous exacerbation of ascites and pleural effusion was noted in 17 (22.1%) of the 77 patients, and these factors were associated significantly (*P* > 0.05). In contrast, no association was noted between exacerbation of ascites and venous thrombus or between exacerbation of pleural effusion and venous thrombus. The 1- and 2-year survival rates were 94.3 and 79.2%, respectively (Figure 1). Regarding the relationship between each factor and the survival rate, significant differences were noted in the Child-Pugh classification and the presence or absence of HCC (*P* = 0.023 and 0.0005, resp.,) (Table 6). On multivariate analysis, the hazard ratio was 6.27 (95% CI: 2.075–19.009) in the Child-Pugh class B/C group and 3.0136 (95% CI: 1.236–7.349) in the group with HCC complications, showing that these were independent factors (Table 7).

## 4. Discussion

The incidence of ascites exacerbation after BRTO has been reported to be low (0–10%) [2, 7, 9, 18], but the modality and timing of evaluation among these reports varies: exacerbation of ascites was evaluated between 24 hours and 4 weeks after BRTO, varying between facilities, and evaluation has not been standardized. In our study, ascites were exacerbated and increased in 33.8% (26/77) within a short time (2 weeks) after BRTO. Cho et al. [8] also reported that ascites was noted on CT in 82% within one week after BRTO, suggesting that the frequency of ascites exacerbation is high immediately to 2 weeks after BRTO. In previous reports on portal hemodynamics [5, 6, 19], portal blood pressure rose significantly immediately after BRTO and then decreased slowly to the baseline 4 weeks after BRTO. Hypoalbuminemia and a high Child-Pugh score were extracted as factors involved in the exacerbation of ascites, and were noted in patients with advanced hepatic cirrhosis. Based on these findings, it was assumed that the addition of temporary portal hypertension to poor liver function exacerbated the ascites, but appropriate evaluation may be difficult when the treatment outcome is only evaluated several months after BRTO due to the combined progression of hepatic cirrhosis-associated ascites. On the other hand, ascites improved after BRTO in one patient. An increase in serum albumin is one factor related to improving ascites. It has also been reported that occlusion of shunts by BRTO increased portal blood flow and resulted in mid- to long-term improvement of liver function [5, 20]. However, in general, albumin levels have been reported to decrease one month after BRTO and it is unlikely that improvement of ascites within 2 weeks after BRTO, as was observed in this patient, would have been due to improvement of the serum albumin levels. Indeed, in this patient, the Child-Pugh class was C, showing poor liver function, and the albumin levels at the time-points of BRTO and ascites evaluation by CT were 2.1 (g/dL) and 2.0 (g/dL), respectively. Thus, the mechanism underlying the influence of BRTO on ascites is complex [21], and further elucidation is necessary.

The reported incidence of pleural effusion after BRTO is in the range of 7%–71% of these patients [7, 11, 13, 18]. In our study, pleural effusion was exacerbated after BRTO in 40.3% (31/77), and it was significant in the patients with hypoalbuminemia. Arai et al. [13] observed a significant difference in the EO dose injected as a sclerosing agent in patients in whom pleural effusion appeared early after BRTO, and concluded that the cause was pulmonary infarction or pulmonary edema induced by EO through vascular shunts. Regarding the pleural effusion that appears following esophageal varix treatment, Kayama et al. [22] suggested that it was related to the EO dose used, and Bacon et al. [23] suggested that the cause was EO-induced inflammation of the mediastinal pleura. Unfortunately, we detected no significant relationship between the EO dose and the development of pleural effusion (data not shown, *P* = 0.809), but the incidence of pleural effusion after BRTO was high. In addition, the incidence of left pleural effusion alone was high (80.7%) among the patients in whom pleural effusion was noted, which could be important. As reported previously, while one possible cause of the exacerbation of pleural effusion was pulmonary infarction and pulmonary edema, considering that the exacerbation of the pleural effusion was unilateral, it could also be possible that the main cause of the exacerbation of pleural effusion was inflammation of the pleura induced by EO influx into the left inferior phrenic, pericardiac, and innominate veins and many anastomotic branches continuous to the gastrorenal shunts that are distributed around the diaphragm. In addition, exacerbation of ascites and pleural effusion was noted simultaneously in 17 (22.1%) of the 77 patients, and these were associated significantly. Furthermore, the exacerbation of pleural effusion was significant in patients with hypoalbuminemia, suggesting that it could serve as an exacerbation factor of ascites, as well as pleural effusion. EO immediately binds albumin in the blood, resulting in its inactivation [24]. It is necessary to further investigate the influence of EO as a sclerosing agent on the lung and whole body, when free EO enters the systemic circulation in hypoalbuminemia. 

Venous thrombus was noted in 9.1% (7/77) of the patients, suggesting that this is a relatively frequent complication. No causal relationship between venous thrombus formation and liver function, ascites, or pleural effusion was noted. The main cause of this venous thrombosis may be intravascular injury due to poor catheter procedure or impairment of vascular endothelium by leakage of EO from the draining or feeding vessel [25]. Cho et al. [11, 12] also reported that venous thrombus was noted after BRTO on CT in 15% (9/60), showing that this is a frequent complication. There have been no other previous reports that have described venous thrombus after BRTO in detail, and only the present study has evaluated the presence or absence of venous thrombus by CT as an early complication, within 2 weeks after BRTO. Since the thrombus later disappeared in 4 patients who could be followed, it is unlikely that venous thrombus that had appeared early after BRTO was involved in the later severe complication and survival, but it should be recognized as a BRTO-associated complication that occurs at a relatively high incidence. 

According to preceding reports [3, 9], exacerbation of collateral veins can occur one or more months after BRTO. Miyamoto et al. [6] reported that elevated portal pressure immediately after BRTO returned to baseline within four weeks along with the development of substitutive collateral circulation. In our study, exacerbation of esophageal varices was noted on GF within one month after BRTO in 25.8% (14/66), but no remarkable exacerbation, that is, from *F0* to *F2/3* or from *F1* to *F3*, was noted. Of the 14 cases of esophageal variceal exacerbation on GF, only one case could be confirmed by CT. Cho et al. investigated the appearance of collateral veins immediately after BRTO using CT and observed no new collateral vein or development of existing collateral vein. Based on these findings, the influence of portal blood pressure elevation immediately after BRTO on esophageal varices may not be significant enough to be detected as wall thickening on CT. 

When factors contributing to worsening the prognosis, including ascites, pleural effusion, and venous thrombus observed, immediately after BRTO, were investigated by multivariate analysis using the Cox proportional hazards model, Child-Pugh classes excluding A and complication of HCC were reported as prognostic factors. This result was consistent with those in preceding reports [2, 3]. 

In conclusion, ascites, pleural effusion, and esophageal varices may become exacerbated within a short time after BRTO. It was suggested that the ascites was exacerbated by hypofunction of the liver and rapid development of portal hypertension after BRTO, and the direct cause of the exacerbation of pleural effusion was EO, which was used as a sclerosing agent. However, the exacerbations of pleural effusion and ascites were associated, and these may have been due to hypoalbuminemia; further supporting that the details of the mechanism are still unclear. However, it was suggested that ascites, pleural effusion, and venous thrombus, which appear early after BRTO, are not prognosis-exacerbating factors.

## Figures and Tables

**Table 1:** Characteristics before BRTO.

Age (years)	61.6 ± 12.2
Gender	
Male	51
Female	26
Timing of treatment	
Emergency cases	15
Prophylactic cases	62
Etiology	
HBV	3
HCV	36
Alcohol	26
NASH	4
PBC	3
AIH	2
IPH	3
Serum albumin (g/dL)	3.2 ± 0.6
Serum total bilirubin (mg/dL)	1.3 ± 0.7
Prothrombin time (%)	72.7 ± 13.2
Plateles (×10^4^ *μ*L)	9.2 ± 3.7
Child-Pugh	
Score	7.2 ± 1.7
Classification	
A (5-6)	32
B (7–9)	37
C (10–15)	8
Esophageal varices	
Present	44
Absent	24
Unknown	9
Hepatocellular carcinoma	
Present	30
Absent	47

Values expressed as mean ± standard deviation.

**Table 2:** Short-term effects and early complications after BRTO.

	Present	Absent	**%**
CT			
Ascites	26	51	33.8
Pleural effusion	31	46	40.3
Location L/R/B	25/1/5		80.7/3.2/16.1
Thrombus	7	70	9.1
Location PV/SPV/Lt.RV	3/2/2		43.0/28.5/28.5
Endoscopy			
Worsening of esophageal varices	14	52	21.2
(unknown: 11)			

PV: portal vein, SPV: splenic vein, Lt.RV: left renal vein.

**Table 3:** Comparison of demographic variables between the patients with and without ascites after BRTO.

Ascites	Yes (*n* = 26)	No (*n* = 51)	*P* value
Age (years)	64.2 ± 10.6	60.2 ± 12.8	0.1778
Gender			0.3666
Male	19	32	
Female	7	19	
Timing of treatment			0.2439
Emergency cases	7	8	
Prophylactic cases	19	43	
Etiology			0.9351
Viral infection	13	26	
No viral infection	13	25	
Serum albumin (g/dL)	2.95 ± 0.47	3.36 ± 0.61	0.0067*
Serum total bilirubin (mg/dL)	1.53 ± 0.92	1.12 ± 0.55	0.0342*
Prothrombin time (%)	66.88 ± 12.43	75.71 ± 12.71	0.0076*
Platelets (×10^4^ *μ*L)	8.13 ± 2.89	9.68 ± 4.03	0.0915
Child-Pugh score	8.08 ± 1.60	6.76 ± 1.57	0.0023*
Hepatocellular carcinoma			0.9488
Present	10	20	
Absent	16	31	
CT findings			
Pleural effusion	17	14	0.0013*
Thrombus	2	5	0.7611

*P < 0.05.

**Table 4:** Comparison of demographic variables between the patients with and without pleural effusion after BRTO.

Pleural effusion	Yes (*n* = 31)	No (*n* = 46)	*P* value
Age (years)	63.9 ± 11.2	60.0 ± 12.6	0.1631
Gender			0.7937
Male	20	31	
Female	11	15	
Timing of treatment			0.5737
Emergency cases	7	8	
Prophylactic cases	24	38	
Etiology			0.8896
Viral infection	16	23	
No viral infection	15	23	
Serum albumin (g/dL)	3.05 ± 0.44	3.34 ± 0.66	0.0412*
Serum total bilirubin (mg/dL)	1.40 ± 0.99	1.17 ± 0.44	0.1742
Prothrombin time (%)	71.00 ± 13.09	73.89 ± 13.31	0.3459
Platelets (×10^4^ *μ*L)	8.37 ± 2.76	9.69 ± 4.22	0.1367
Child-Pugh score	7.58 ± 1.48	6.96 ± 1.79	0.1144
Hepatocellular carcinoma			0.9704
Present	12	18	
Absent	19	28	
CT findings			
Ascites	17	9	0.0019*
Thrombus	4	3	0.3479

*P < 0.05.

**Table 5:** Comparison of demographic variables between the patients with and without thrombus development in the major systemic and portal vein after BRTO.

Thrombus	Yes (*n* = 7)	No (*n* = 70)	*P* value
Age (years)	57.4 ± 12.3	61.9 ± 12.2	0.3480
Gender			0.7937
Male	5	46	
Female	2	24	
Timing of treatment			0.7175
Emergency cases	1	14	
Prophylactic cases	6	56	
Etiology			0.6666
Viral infection	3	36	
No viral infection	4	34	
Serum albumin (g/dL)	2.8 ± 0.49	3.27 ± 0.59	0.0566
Serum total bilirubin (mg/dL)	1.34 ± 0.59	1.25 ± 0.73	0.7470
Prothrombin time (%)	65.57 ± 13.55	73.44 ± 13.06	0.1370
Platelets (×10^4^ *μ*L)	9.48 ± 2.43	9.12 ± 3.85	0.8062
Child-Pugh score	8.28 ± 1.70	7.10 ± 1.66	0.0873
Hepatocellular carcinoma			0.9704
Present	3	27	
Absent	4	43	
CT findings			
Ascites	2	24	0.7611
Pleural effusion	4	27	0.3479

**Table 6:** Univariate analysis of prognostic factors affecting overall survival after BRTO.

Variable	*P* value
Age (≥70 y)	0.2667
Gender (male)	0.2714
Emergency cases	0.1547
Etiology (viral)	0.3892
Alb (≥3.0 g/dL)	0.1371
T-bil (≥1.4 mg/dL)	0.8149
PT (≥70%)	0.9645
Platelets (≥8.0 × 10^4^ *μ*L)	0.6529
Child-Puge classification B/C	**0.023**
HCC (present)	**0.0005**
Esophageal varices (present)	0.5296
Ascites (present)	0.095
Pleural effusion (present)	0.064
Thrombus (present)	0.8823

Alb: serum albumin, T-bil: serum total bilirubin, PT: prothrombin time, HCC: hepatocellular carcinoma.

**Table 7:** Multivariate analysis of prognostic factors affecting overall survival after BRTO.

Variable	RR (95% CI)	*P* value
Child-Puge classification B/C	6.2798 (2.0746–19.0094)	0.028
HCC (present)	3.0136 (1.2358–7.3491)	0.004

HCC: hepatocellular carcinoma.

## References

[1] Kanagawa H, Mima S, Kouyama H, Gotoh K, Uchida T, Okuda K (1996). Treatment of gastric fundal varices by balloon-occluded retrograde transvenous obliteration. *Journal of Gastroenterology and Hepatology*.

[2] Fukuda T, Hirota S, Sugimura K (2001). Long-term results of balloon-occluded retrograde transvenous obliteration for the treatment of gastric varices and hepatic encephalopathy. *Journal of Vascular and Interventional Radiology*.

[3] Ninoi T, Nishida N, Kaminou T (2005). Balloon-occluded retrograde transvenous obliteration of gastric varices with gastrorenal shunt: long-term follow-up in 78 patients. *American Journal of Roentgenology*.

[4] Koito K, Namieno T, Nagakawa T, Morita K (1996). Balloon-occluded retrograde transvenous obliteration for gastric varices with gastrorenal or gastrocaval collaterals. *American Journal of Roentgenology*.

[5] Akahane T, Iwasaki T, Kobayashi N (1997). Changes in liver function parameters after occlusion of gastrorenal shunts with balloon-occluded retrograde transvenous obliteration. *American Journal of Gastroenterology*.

[6] Miyamoto Y, Oho K, Kumamoto M, Toyonaga A, Sata M (2003). Balloon-occluded retrograde transvenous obliteration improves liver function in patients with cirrhosis and portal hypertension. *Journal of Gastroenterology and Hepatology*.

[7] Shimoda R, Horiuchi K, Hagiwara S (2005). Short-term complications of retrograde transvenous obliteration of gastric varices in patients with portal hypertension: effects of obliteration of major portosystemic shunts. *Abdominal Imaging*.

[8] Cho SK, Shin SW, Yoo EsY (2007). The short-term effects of balloon-occluded retrograde transvenous obliteration, for treating gastric variceal bleeding, on portal hypertensive changes: a CT evaluation. *Korean Journal of Radiology*.

[9] Chikamori F, Kuniyoshi N, Shibuya S, Takase Y (2001). Eight years of experience with transjugular retrograde obliteration for gastric varices with gastrorenal shunts. *Surgery*.

[10] Arai H, Abe T, Takagi H, Mori M (2006). Efficacy of balloon-occluded retrograde transvenous obliteration, percutaneous transhepatic obliteration and combined techniques for the management of gastric fundal varices. *World Journal of Gastroenterology*.

[11] Cho SK, Shin SW, Lee IH (2007). Balloon-occluded retrograde transvenous obliteration of gastric varices: outcomes and complications in 49 patients. *American Journal of Roentgenology*.

[12] Cho SK, Shin SW, Do YS (2008). Development of thrombus in the major systemic and portal veins after balloon-occluded retrograde transvenous obliteration for treating gastric variceal bleeding: its frequency and outcome evaluation with CT. *Journal of Vascular and Interventional Radiology*.

[13] Arai H, Abe T, Takayama H (2011). Respiratory influences of balloon occluded retrograde transvenous obliteration of gastric varices: a prospective controlled study. *Journal of Gastroenterology and Hepatology*.

[14] Hirota S, Matsumoto S, Tomita M, Sako M, Kono M (1999). Retrograde transvenous obliteration of gastric varices. *Radiology*.

[15] Takahashi K, Yamada T, Hyodoh H (2001). Selective balloon-occluded retrograde sclerosis of gastric varices using a coaxial microcatheter system. *American Journal of Roentgenology*.

[16] Kiyosue H, Mori H, Matsumoto S, Yamada Y, Hori Y, Okino Y (2003). Transcatheter obliteration of gastric varices: part 1. Anatomic classification. *Radiographics*.

[17] Kiyosue H, Mori H, Matsumoto S, Yamada Y, Hori Y, Okino Y (2003). Transcatheter obliteration of gastric varices: part 2. Strategy and techniques based on hemodynamic features. *Radiographics*.

[18] Sonomura T, Sato M, Kishi K (1998). Ballon-occluded retrograde transvenous obliteration for gastric varices: a feasibility study. *CardioVascular and Interventional Radiology*.

[19] Chikamori F, Kuniyoshi N, Shibuya S, Takase Y (2000). Short-term hemodynamic effects of transjugular retrograde obliteration of gastric varices with gastrorenal shunt. *Digestive Surgery*.

[20] Nakano R, Iwao T, Oho K, Toyonaga A, Tanikawa K (1997). Splanchnic hemodynamic pattern and liver function in patients with cirrhosis and esophageal or gastric varices. *American Journal of Gastroenterology*.

[21] Fukuda T, Hirota S, Matsumoto S (2004). Application of balloon-occluded retrograde transvenous obliteration to gastric varices omplicating refractory ascites. *CardioVascular and Interventional Radiology*.

[22] Kayama H, Inamori M, Togawa JI (2006). Pleural effusions following endoscopic injection sclerotherapy for cirrhotic patients with esophageal varices. *Hepato-Gastroenterology*.

[23] Bacon BR, Bailey-Newton RS, Connors AF (1985). Pleural effusions after endoscopic variceal sclerotherapy. *Gastroenterology*.

[24] Hoak JC, Warner ED, Connor WE (1967). Platelets, fatty acids and thrombosis. *Circulation Research*.

[25] Evans DMD, Jones DB, Cleary BK, Smith PM (1982). Oesophageal varices treated by sclerotherapy: a histopathological study. *Gut*.

